# Ability of the Short Physical Performance Battery Frailty Index to Predict Mortality and Hospital Readmission in Patients with Liver Cirrhosis

**DOI:** 10.1155/2019/8092865

**Published:** 2019-05-02

**Authors:** Mervat Essam Behiry, Sherif Mogawer, Ahmed Yamany, Maha Rakha, Rana Awad, Nahla Emad, Yasmine Abdelfatah

**Affiliations:** School of Medicine, Cairo University, Egypt

## Abstract

**Background/Aims:**

Unplanned hospitalisation is a marker of poor prognosis and a major financial burden in patients with cirrhosis. Frailty-screening tools could determine the risk for unplanned hospital admissions and death. The study aims to evaluate the bedside frailty-screening tool (Short Physical Performance Battery (SPPB)) in prediction of mortality in patients with liver cirrhosis.

**Methods:**

One hundred forty-five patients with liver cirrhosis were recruited from Cairo University Hospital. Clinical assessment and routine laboratory tests were performed, and the SPPB frailty index, Child score, and model for end-stage liver disease (MELD) score were calculated on admission. These metrics were compared to assess mortality outcomes over the course of 90 days.

**Results:**

The mean age of the patients was 60 ± 7 years, and frailty index score (SD) was 6 ± 3. The overall 90-day readmission rate was 43.4%, while the overall 90-day mortality rate was 18.6%. SPPB scores differed significantly between survivors (4.1 ± 1.4) and nonsurvivors (6.47 ± 2.8) (P value ≤ 0.001) as well as between readmitted patients (7.5 ± 2.9) and patients who were not readmitted (4.5 ± 1.9) (P value ≤ 0.001), while the Child and MELD scores showed no associations with patient outcomes. SPPB performed better with a specificity of 72.3% and a sensitivity of 72.2% for predicting mortality.

**Conclusions:**

SPPB could be a screening tool used to detect frailty and excelled over traditional scores as a predictor of death. A low SPPB frailty score among hospitalised patients with cirrhosis is associated with poor outcomes.

## 1. Introduction

Frailty is defined by decreased strength, power, and diminished physiological function that in turn leads to increased physical dependency and increased risk of mortality especially in older age and those with debilitating diseases [1].

Posthepatitic liver cirrhosis has a high prevalence in Egypt. A comprehensive assessment of hepatitis C virus (HCV) epidemiology was conducted in 2018, revealing high incidence and prevalence levels across all populations in Egypt. The pooled mean HCV prevalence was estimated to be 11.9% in the general population, 55.6% among populations at high risk, 14.3% among populations at intermediate risk, and 56.0% among populations with liver-related conditions including liver cirrhosis [2].

HCV-related cirrhosis is strongly associated with protein energy malnutrition (PEM), sarcopenia, frailty, and physical atrophy. This was found to be caused by the release of muscle-wasting cytokines, the derangement of muscle proteins, and the increased autophagy of muscles, all of which are mediated by elevated levels of tumour necrosis factor, elevated concentrations of ammonia, and impaired ureagenesis [3].

The assessment of physical frailty in patients who have undergone liver transplantation has been widely discussed. A strong association between frailty and poor outcomes after transplantation has been reported [4]. The six-minute walk test has been indicated to be a surrogate test for the pretransplant evaluation of functional capacity and a significant determinant of posttransplantation survival [5].

Unplanned hospitalisation is a major risk factor for poor prognosis and increased. Frailty is considered an independent predictor of unplanned hospitalisations or death in cirrhotic outpatients [6].

Given the importance of the detection of frailty and early intervention in cirrhotic patients, many studies have addressed the potential effects of interventional exercise and nutritional supplement strategies [7–9]. These strategies may improve physical function and quality of life and accordingly the frailty index. This would likely decrease the possibility of cirrhosis-associated morbidities, unplanned hospital readmission, health care-related costs, and death [10].

Multiple clinical models of frailty have been proposed that use combinations of different parameters such as the Clinical Frailty Scale (CFS) [11], the Model for End-Stage Liver Disease-Sodium score [12], the activities of daily living (ADL) score, the Braden Scale [13], and the Fried Frailty Index [14]. The Short Physical Performance Battery (SPPB) has emerged as one of the most promising tools for evaluating functional capability. It has proven to provide standard parameters that can be used uniformly across clinical and research settings with high predictiveness for disability onset and adverse outcomes, especially in older patients [15].

In our study, the main objective was to screen for and evaluate frailty among cirrhotic Egyptian patients using the SPPB and to determine the impact of frailty on hospital readmission and mortality.

## 2. Methods

### 2.1. Study Design and Sample Population

Our cohort study included 145 Egyptian patients with liver cirrhosis. This study was conducted at Kasr Al-Aini Hospital. Patients with posthepatitic cirrhosis who were ≥ 18 years old were selected from the internal medicine wards, while patients with current hepatic or extrahepatic malignancies; patients with overt hepatic encephalopathy; patients in comas; patients with any medical, physical, neurological disabilities; and patients who used medications (sedatives and anticonvulsants) that compromised their balance were excluded from the study.

Full history and clinical examination were done for all patients.

#### 2.1.1. Anthropometric Assessments

Weight and body mass index (BMI) were measured for each patient.

#### 2.1.2. Blood Collection and Sample Preparation

Ten millilitres of blood was withdrawn from each subject. The complete blood count was estimated using a cell counter with a Cell Dyn machine. The estimation of the levels of serum creatinine and liver enzymes was performed using a kinetic method via an automated Dimension system. The serum levels of albumin, prothrombin concentration (PC), the international normalized ratio (INR) for prothrombin time, and the thyroid profile were also determined.

#### 2.1.3. Model End-Stage Liver Disease MELD Score Evaluation

The MELD score uses the patient's serum levels of* bilirubin* and* creatinine* and their INR to predict survival [16]. It is calculated according to the following formula:

MELD = 3.78 × ln[serum bilirubin (mg/dL)] + 11.2 × ln[INR] + 9.57 × ln[serum creatinine (mg/dL)] + 6.43. According to their MELD score “20” patients were classified into the following two groups:

Group (A): patients with MELD scores > 20.

Group (B): patients with MELD scores ≤ 20.

#### 2.1.4. Child-Turcotte-Pugh (CTP) Classification

The CTP score combines five clinical measures of liver disease. Each measure is scored from 1 to 3, with 3 indicating the most severe level of derangement. Patients with chronic liver disease are classified as Child–Pugh classes A to C [17].

#### 2.1.5. Frailty Assessment according to the Short Physical Performance Battery (SPPB)

The SPPB is a functional test that measures gait speed (8-foot walk), standing balance, and lower extremity strength and power (via a task involving rising from a chair). The average of three trials was used. Each test was scored on a scale from 0 to 4 points, with a total score range of 0 to 12 points [18]. The patients were contacted after three months to determine the outcomes of mortality or readmission to the hospital.

#### 2.1.6. Handgrip Assessment

The purpose of this test is to measure the maximum isometric strength of the hand and forearm muscles. Each subject holds a handgrip dynamometer (Lafayette, USA) in his /her hand, with the arm at a right angle to his/her body and the elbow held by their side. The best of three attempts, with 30 seconds of rest between the trials, for each hand was recorded in kilograms to one decimal point [19].

### 2.2. Data Management and Statistical Analysis

Data were precoded and entered in Microsoft Excel. Quantitative variables are presented as the mean (SD). Qualitative variables are described as numbers and percentages. A chi-square test was used to compare qualitative variables between groups. An unpaired t-test was used to compare quantitative variables in the parametric data (SD<50% mean). A paired t-test was used to compare quantitative variables within the same group. A P value < 0.05 was considered significant. Pearson's correlation was used to test for statistically significant associations. The data have been tabulated for visualization.

### 2.3. Ethical Considerations

Written informed consent was obtained from all subjects. The study protocol maintained patient confidentiality and conformed to the standards of the Declaration of Helsinki; the study protocol was revised, accepted, and approved by the internal review board (Ethical Committee of Internal Medicine, Faculty of Medicine, Cairo University, protocol number* 16-2017-/2018 on the date of *26/8/2017).

## 3. Results

### 3.1. Demographic and Laboratory Data

In our study, the mean age of the patients was 60 ± 7 years, ranging from 36 to 80 years. Seventy-five out of 145 patients were male. The mean (SD) MELD score was 16 ± 6, while the mean CTP score was 10 ± 2. The demographics, clinical characteristics, and laboratory data are shown in Table 1.

### 3.2. Readmission and 90-Day Mortality

The overall 90-day readmission rate was 43.4% and it mainly occurred due to cirrhosis-related complications, including hematemesis (28 patients), in addition to hepatic coma (19 patients), ascites necessitating tapping (10 patients), and comorbidities-related (8 patients). The overall 90-day mortality reaches 18.6%.

### 3.3. Correlations with the SPPB Frailty Score

The data revealed that there was a significant negative correlation between the frailty score assessed by the SPPB and age, CTP score, and MELD score (r=-0.428, -0.509, and -0.262 and p=0.001, 0.001, and 0.047, respectively). Frailty had a positive correlation with the handgrip test score (r=0.568 and p=0.001), as shown in Table 2.

The results demonstrated that the overall 90-day readmission occurred in patients with mean frailty score of 4.88 ± 1.96, MELD score of 16.18 ± 6.72, CTP score of 9.88 ± 1.55, and handgrip score of 14.012 ± 5.7, and those with the overall 90-day mortality had mean frailty score of 4.18 ± 1.47, MELD score of 17.27 ± 9.5, CTP score of 9.64 ± 1.28, and handgrip score of 12.909 ± 5.39

The frailty score was significantly higher in males (6.83 ± 2.866) than in females (5.18 ± 2.420) (p = 0.021). The frailty score was 4.88 ± 1.96 in patients who were readmitted to the hospital, compared with 7.56 ± 2.95 in those who were not readmitted to the hospital (p value < 0.001). Survivors had significantly higher frailty scores than nonsurvivors (6.47 ± 2.8* versus *4.18 ± 1.47, respectively; p ≤ 0.001); however frailty scores did not differ between patients with and without comorbidities.

The data showed that patients with lower frailty scores (4.88 ± 1.965) had a higher risk of hospital readmission than those with higher scores (7.56 ± 2.959) (p ≤ 0.001).

Ninety-day mortality was associated only with older age; the mean age of survivors was 59.17 ± 6.907 years, while the mean age of nonsurvivors was 64.36 ± 7.474 years (p value = 0.031). However, no significant differences in mortality were observed with regard to sex, BMI, the presence of comorbidities, or laboratory profiles.

Hospital readmission was not associated with any demographic, laboratory, or clinical parameters.

There was no significant difference in patient survival and hospital readmission based on MELD, CTP, or handgrip scores.

### 3.4. The Sensitivity and Specificity of Frailty Score in Prediction of Increased Mortality and 90-Day Readmission

**Figure 1:**
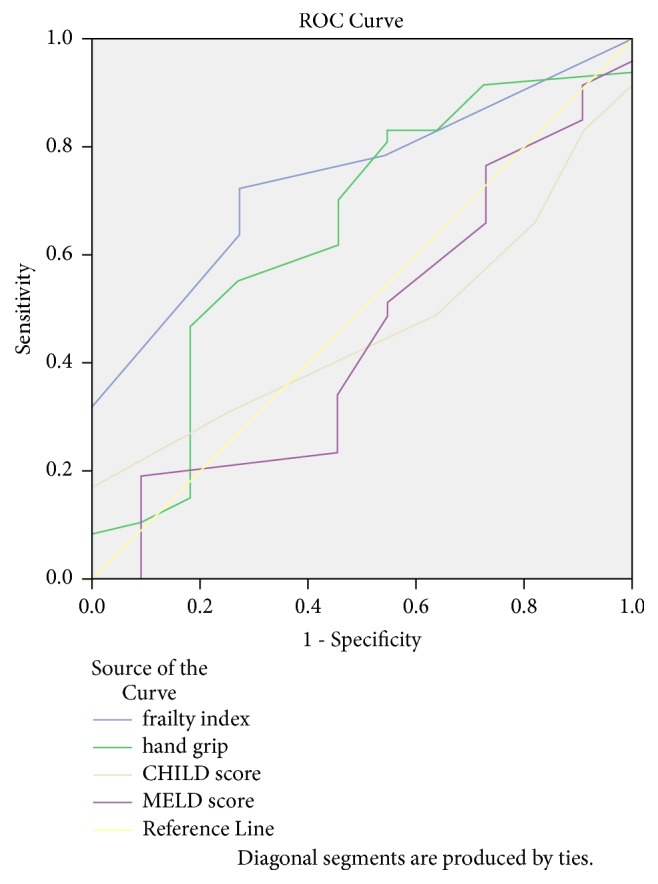
ROC curve for mortality predictors among inpatients with liver cirrhosis.

The ROC curve analyses of mortality based on frailty, handgrip, MELD, and CTP scores revealed that only frailty score had a significant area under the curve (AUC) (0.743; p value = 0.013). Frailty scores had fair sensitivity and specificity (72.7% and 72.3%, respectively) at a criterion of 4.50 with a 95% CI of 0.603-0.883 as shown in Figure 1.

However none of the before-mentioned parameters were potential predictors of hospital readmission (Table 3).

## 4. Discussion

The current results revealed that a lower frailty score was associated with hospital readmission and mortality. The objective of the current study was to screen for frailty among cirrhotic hospitalised patients and evaluate the role of frailty in unplanned hospital readmission and increased mortality.

There are established models for predicting the risk of poor outcomes such as CTP and MELD scores, yet their discriminative abilities are controversial [20]. Moreover, the frailty assessment is not included [21]. Factors associated with increased sarcopenia and cirrhosis include older age, increased severity of the associated liver disease, the presence of other chronic comorbidities, and longer duration of end-stage liver disease [22].

Frailty mainly contributes to malnutrition, which is prevalent in 60% of end-stage liver disease patients. This is due to poor dietary intake, anorexia, fat malabsorption-associated disorders such as chronic pancreatitis, and disrupted hepatic metabolism [23, 24].

In this study, the SPPB was used to assess frailty. The benefits of this assessment tool include good reliability, validity, and responsiveness as well as simplicity. In addition, the SPPB only requires 5 to 10 minutes to complete, so it can be integrated into patient management without excessive time consumption [25].

In the present study, the mean (SD) score on the SPPB, which combines the results of gait speed, chair standing, and balance tests, was 6 ± 3. There was a significant negative correlation between frailty as assessed by the SPPB and age, CTP scores, and MELD scores. Frailty index was positively correlated with the handgrip score. Dunn and colleagues reported that, for each 0.10-m/sec reduction in walking speed, there is a 22% increase in the number of hospitalised days in frail patients with cirrhosis [26].

The reported readmission estimates were variable, with heterogeneous findings [27] ranging from 10% to 71% [28–30]. It has been noted that there is a robust relationship between readmission and subsequent mortality [31–33]. The overall 90-day readmission rate was 43.4%, while the overall 90-day mortality rate was 18.6%, and the most common cause of readmission in our population was cirrhosis-related complications. Haematemesis was the major cause of readmission, followed by hepatic encephalopathy [27]. Few studies have noted that increased readmission among cirrhotics is more often seen in those with diabetes. Diabetes is associated with a greater than 70% readmission rate due to increased incidence of infections and renal impairment [34, 35].

In the current study, the frailty scores were lower in patients with unplanned hospital readmission and in nonsurvivors. Ninety-day mortality was associated only with older age. There were no significant differences in mortality with regard to sex, BMI, the presence of comorbidities, or laboratory profiles. Hospital readmission was not associated with any demographic, laboratory, or clinical parameters.

Although MELD and CTP scores are disease-specific measurements that are commonly used in the care of patients with cirrhosis, neither MELD nor CTP scores differed between survivors and nonsurvivors or between patients who were readmitted to hospital and those who were not. This finding might be explained by the fact that the MELD score depends mainly on dynamic parameters that could be affected by laboratory variation. Some variables of the CTP score (hepatic encephalopathy, ascites grade, and nutrition) are subjective [13]. In addition, this study had a relatively small sample size, and most of our study population had MELD scores less than “20”, reducing the comparative and discriminative abilities of this study.

Attempting to assess predictors of poor outcome, the current study examined the performance of the SPPB, handgrip test, MELD, and CTP. The results revealed that the frailty score is a potential predictor of mortality in cirrhotic patients, with fair sensitivity and specificity and its performance was superior to that of the handgrip test. The SPPB was found to be better at assessing physical function than the handgrip test possibly because it involves more complex coordination and depends on a larger portion of the total body muscle mass.

Regarding hospital readmission, none of the aforementioned measurements affected the 90-day risk of readmission. This finding was contradictory to those of other studies, which demonstrated an association between the gait speed portion of the frailty test and subsequent hospitalisation in elderly people [36–38]. This difference might be attributed to the variation in the characteristics of the different study populations that may have affected their readmission.

The limitations of this study include the small sample size. Secondly, sarcopenia was not assessed, which may infer a causal relation with frailty. However, this study points to an area of future inquiry in our cirrhotic patients.

We recommend further studies with larger populations and the construction of a combined model that merges the different scores and parameters to increase accuracy and precision.

To conclude, frailty is easily assessed by the SPPB which does not require extensive training for clinicians or nurses. A low SPPB frailty score among hospitalised patients with cirrhosis is associated with poor outcomes. The SPPB could serve as a predictor of hospital readmission and increased mortality among cirrhotic patients.

## Figures and Tables

**Table 1:** Demographic, clinical, and laboratory data of the studied population.

Variables	N=145
Age (mean ± SD^*∗*^)	60 ± 7

Gender (N, %)	
Male	75(51.7%)
Female	70(48.3%)

Body mass index (mean ± SD)	24.2 ± 3.4

CPT† score (mean ± SD)	10 ± 2
Class (A)	12(8.3%)
Class(B)	50(34.5%)
Class(C)	83(57.2%)

MELD‡ Score (mean ± SD)	16 ± 6
MELD > 20	15(10%)
MELD ≤ 20	130(90%)

Comorbidities (N,%)	
Diabetes Mellitus	45(31%)
Hypertension	22(15.2%)

Hematemesis (N, %)	28(19.3%)

Hb^#^ gm/dl (mean ± SD)	9.11 ± 1.71

Albumin gm/dl (mean ± SD)	2.4 ± 0.5

Creatinine mg/dl (mean ± SD)	1.4 ± 0.3

Total Bilirubin mg/dl (mean ± SD)	1.7 ± 0.9

ALT^§^ IU/L (mean ± SD)	51 ± 97

AST^¶^ IU/L (mean ± SD)	92 ± 198

INR^††^ (mean ± SD)	1.61 ± 0.91

TSH^*∗∗*^ IU/L (mean ± SD)	1.99 ± 1.3

CrP^‡‡^ (mg/dl)	22.6

Hand grip score	14.9 ± 5.6

Short Physical Performance Battery	6 ± 3

Hospital readmission (3 months)	63(43.4%)

Patient survival (3 months)	
Survivors (N, %)	118(81.4%)
Non-survivors (N, %)	27(18.6%)

^*∗*^SD standard deviation: *∗*, †, ‡, §, II, ¶, and #.

^†^CPT: Child-Turcotte-Pugh; ^‡^MELD: model for end stage liver disease; ^§^ALT: alanine transaminase; ^¶^AST: aspartate transaminase; ^#^Hb: hemoglobin; ^*∗∗*^TSH: thyrotropin stimulating hormone; ^††^INR: international normalized ratio; ^‡‡^CrP: C reactive protein.

**Table 2:** Correlation between Frailty score and other parameters.

Variable	Frailty score	
	R-value	p-value
Age	-0.428	0.001

BMI^*∗*^	0.134	0.315

Hemoglobin (Hb%)	0.065	0.630

Albumin	0.333	0.011

ALT^†^	0.058	0.664

AST^‡^	0.088	0.512

CrP^§^	-0.182	0.173

Hand grip	0.568	0.001

CTP^¶^ score	-0.509	0.001

MELD^#^ score	-0.262	0.047

^*∗*^BMI: body mass index; ^†^ALT: alanine transaminase; ^‡^AST: aspartate transaminase; ^§^CrP: C reactive protein; ^¶^CPT: Child-Turcotte-Pugh; ^#^MELD: model for end stage liver disease.

**Table 3:** Results of ROC curve analyses of predictors of hospital readmission.

Variable(s)	AUC^*∗*^	Cut-off point	P value	Sensitivity	Specificity	PPV	NPV	Asymptotic 95% Confidence Interval	
frailty index	0.383	5.50	0.136	45.5%	58.3%	40.0%	63.6%	0.236	0.529

hand grip	0.460	14.50	0.614	45.5%	47.2%	34.5%	58.6%	0.302	0.619

CTP^†^ score	0.612	9.50	0.156	40.9%	47.2%	32.1%	56.7%	0.466	0.758

MELD^‡^ score	0.522	15.50	0.779	50.0%	50.0%	37.9%	62.1%	0.367	0.677

^†^AUC: area under curve; ^†^CPT: Child-Turcotte-Pugh; ^‡^MELD: model for end stage liver disease.

## Data Availability

The data used to support the findings of this study are available from the corresponding author upon request.
